# Deep Learning Segmentation of Triple-Negative Breast Cancer (TNBC) Patient Derived Tumor Xenograft (PDX) and Sensitivity of Radiomic Pipeline to Tumor Probability Boundary

**DOI:** 10.3390/cancers13153795

**Published:** 2021-07-28

**Authors:** Kaushik Dutta, Sudipta Roy, Timothy Daniel Whitehead, Jingqin Luo, Abhinav Kumar Jha, Shunqiang Li, James Dennis Quirk, Kooresh Isaac Shoghi

**Affiliations:** 1 Department of Radiology, Washington University School of Medicine, St. Louis, MO 63110, USA; kaushik.dutta@wustl.edu (K.D.); sudiptaroy@wustl.edu (S.R.); tdwhitehead@wustl.edu (T.D.W.); a.jha@wustl.edu (A.K.J.); jdquirk@wustl.edu (J.D.Q.); 2 Department of Surgery, Washington University School of Medicine, St. Louis, MO 63110, USA; jingqinluo@wustl.edu; 3 Department of Biomedical Engineering McKelvey School of Engineering, Washington University in St. Louis, St. Louis, MO 63110, USA; 4 Department of Medicine, Division of Oncology, Washington University School of Medicine, St. Louis, MO 63110, USA; shunqiangli@wustl.edu

**Keywords:** deep learning, segmentation, radiomics, preclinical imaging, triple negative breast cancer, co-clinical imaging

## Abstract

**Simple Summary:**

Co-clinical trials are an emerging area of investigation in which a clinical trial is coupled with a corresponding preclinical trial to inform the corresponding clinical trial. The preclinical arm aids in assessing therapeutic efficacy, patient stratification, and designing optimal imaging strategies. There is much interest in harmonizing preclinical and clinical quantitative imaging pipelines. Radiomics is widely explored in clinical imaging to predict response to therapy. In preclinical imaging, high-throughput radiomic analysis is limited by manual delineation of tumor boundaries, which is labor intensive with poor reproducibility. Our proposed deep-learning-based system was trained to automatically segment tumors from multi-contrast MR images and extract radiomic features. The proposed method is highly reproducible with significant correlation in radiomic features. The deployment of this pipeline in the preclinical arm would provide high throughput and reproducible radiomic analysis.

**Abstract:**

Preclinical magnetic resonance imaging (MRI) is a critical component in a co-clinical research pipeline. Importantly, segmentation of tumors in MRI is a necessary step in tumor phenotyping and assessment of response to therapy. However, manual segmentation is time-intensive and suffers from inter- and intra- observer variability and lack of reproducibility. This study aimed to develop an automated pipeline for accurate localization and delineation of TNBC PDX tumors from preclinical T1w and T2w MR images using a deep learning (DL) algorithm and to assess the sensitivity of radiomic features to tumor boundaries. We tested five network architectures including U-Net, dense U-Net, Res-Net, recurrent residual UNet (R2UNet), and dense R2U-Net (D-R2UNet), which were compared against manual delineation by experts. To mitigate bias among multiple experts, the simultaneous truth and performance level estimation (STAPLE) algorithm was applied to create consensus maps. Performance metrics (F1-Score, recall, precision, and AUC) were used to assess the performance of the networks. Multi-contrast D-R2UNet performed best with F1-score = 0.948; however, all networks scored within 1–3% of each other. Radiomic features extracted from D-R2UNet were highly corelated to STAPLE-derived features with 67.13% of T1w and 53.15% of T2w exhibiting correlation ρ ≥ 0.9 (*p* ≤ 0.05). D-R2UNet-extracted features exhibited better reproducibility relative to STAPLE with 86.71% of T1w and 69.93% of T2w features found to be highly reproducible (CCC ≥ 0.9, *p* ≤ 0.05). Finally, 39.16% T1w and 13.9% T2w features were identified as insensitive to tumor boundary perturbations (Spearman correlation (−0.4 ≤ ρ ≤ 0.4). We developed a highly reproducible DL algorithm to circumvent manual segmentation of T1w and T2w MR images and identified sensitivity of radiomic features to tumor boundaries.

## 1. Introduction

Triple-negative breast cancer (TNBC) is a highly heterogeneous and aggressive cancer characterized by poor outcomes and higher relapse rates compared to other subtypes of breast cancer. Pathological complete response (pCR) is often used as a critical endpoint in the treatment of TNBC following neoadjuvant chemotherapy (NAC) as it is often associated with favorable long-term outcomes. Therefore, it is critical to identify patients who will respond to NAC therapy to avoid the use of ineffective treatments. To support this effort, co-clinical trials are an emerging area of investigation, whereby a clinical trial is coupled with a corresponding preclinical trial to inform the corresponding clinical trial [1,2,3,4,5,6,7]. The emergence of patient-derived tumor xenografts (PDXs) as a co-clinical platform is largely motivated by the realization that established cell lines do not recapitulate the heterogeneity of human tumors and the diversity of tumor phenotypes [8] and that better oncology models are needed to support high-impact translational cancer research [9,10,11].

In this context, preclinical imaging is a critical component in the co-clinical research pipeline, both in academia as well as in industry, to validate imaging biomarkers, to detect disease, and to assess therapeutic efficacy. To that end, T1- and T2-weighted MR images are routinely used to extract morphological and pathological information from tumor lesions. Contrast-enhanced MR is additionally used to derive functional information on tumor perfusion [12,13,14]. In this context, accurate localization and delineation of tumor boundaries is vital for assessing treatment response. Manual segmentation by experts, however, is time- and labor-intensive and suffers from inter- and intra-observer variability along with limited reproducibility. In order to mitigate the observer variability, semi-automatic and automatic methods have been employed to segment tumors, primarily in the clinical research setting with fewer for preclinical applications. Recently, DL algorithms based on convolutional neural network (CNN) have shown efficacy in accurately locating and segmenting tumor boundaries in clinical settings. They outperform other traditional automated algorithms for MR tumor segmentation in clinical settings [15,16,17]. The U-Net [18] architecture is one of the most widely used approaches in medical image segmentation, which involves both encoder and decoder layers along with skip connections. Several variants of the U-Net architecture have been developed, including the residual U-Net (Res-UNet) [19] and the recurrent residual U-Net (R2UNet) [20], for better feature representation and to mitigate the vanishing gradient problem in training deep architecture. 

**Figure 1:**
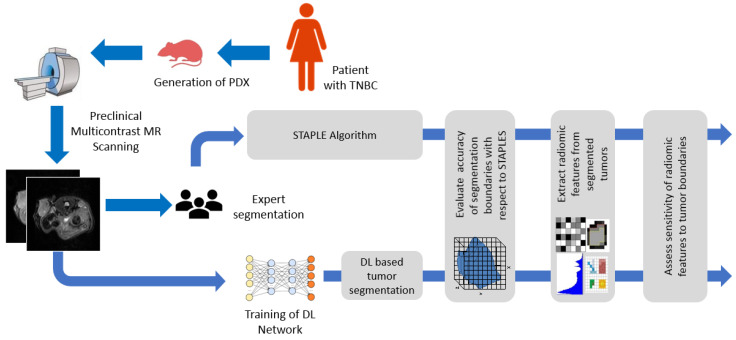
Overview of the pipeline of the project.

The objective of this study was to develop and evaluate the performance of DL-based tumor segmentations algorithm in multi-contrast preclinical MR imaging to alleviate manual effort in tumor segmentation and to circumvent observer variability in tumor delineation. An overview of the proposed pipeline is depicted in Figure 1. U-Net [18], Res-Unet [19,21], and R2Unet [20] architectures were implemented to that end. In addition, recent works have suggested that dense interconnections may alleviate the vanishing gradient problem, strengthen feature propagation, encourage feature reuse, and substantially reduce the number of parameters [22]. For this reason, dense interconnections of U-Net [23] and R2UNet [24] was implemented as well. Advanced quantitative imaging methods, such as radiomics [25], facilitate the extraction of higher dimensional data from the radiological images to characterize tumor heterogeneity and to assess treatment response [26]. To enhance translational insight, the imaging biomarkers derived from radiomic analysis should be robust and reproducible to exhibit clinical relevance. To assess the reproducibility of the features, it is essential to analyze sensitivity of the features to intra- and inter-observer variability arising from manual segmentation. To that end, we extracted radiomic features from the segmented tumor regions to analyze the level of agreement between and within manual and automated methods. In doing so, we examined the sensitivity of the features to tumor boundaries and the reproducibility of the algorithm, signifying its reliability and robustness to that of manual annotation.

## 2. Materials and Methods

### 2.1. Generation of TNBC PDXs

Gene expression analyses of 93 TNBC PDXs (29,657 unique genes/probes) were performed to identify six TNBC subtypes, which included 2 basal-like (BL1 and BL2), an immunomodulatory (IM), a mesenchymal (M), a mesenchymal stem—like (MSL), and a luminal androgen receptor (LAR) subtype [27]. Details regarding animals, surgeries, and tumor xenografts were reported previously [28] and are publicly available at https://c2ir2.wustl.edu/ (accessed on 26 July 2021). All animal experiments were conducted in compliance with the Guidelines for the Care and Use of Research Animals established by Washington University’s Animal Studies Committee.

### 2.2. MR Image Acquisition

MR image acquisition was performed using a MR Solutions small animal simultaneous 7T MR/PET scanner (MR Solutions, Guildford, UK). MR imaging included T1-weighted (T1w) and T2-weighted (T2w) sequences acquired in axial oblique planes perpendicular to the spine of the mouse. PDX mice were anesthetized with 1–2% isoflurane throughout imaging sessions. MR imaging data were obtained for forty-nine mice with TNBC PDX tumors implanted in the inguinal mammary fat pad. The spatial resolution was 0.25 mm × 0.25 mm × 1 mm with a 0.1 mm gap between the slices. The imaging field of view (FOV) was fixed at 32 mm by 24 mm to cover the entire tumor and four repetitions were acquired and averaged for improved SNR and to reduce motion artifacts. For each PDX, 12–16 T1w and T2w trans-axial slices were obtained with an image dimension of 128 × 128, and were retrieved from the scanner in DICOM format. The multi-parametric MR image acquisition protocol was as follows: T1w—2D T1-weighted fast spin echo (FSE) multi-slice images were acquired with echo train length 4, echo spacing 11 ms, effective echo time (TE) = 11 ms, respiratory gated with effective repetition time (TR) = 833 ms, respiration rate of about 60 breaths/min. T2w—2D T2-weighted FSE multi-slice images were obtained with echo train length 4, echo spacing 15 ms, effective echo time (TE) = 45 ms, respiratory gated with effective repetition time (TR) = 5000 ms, respiration rate of about 60 breaths/min. 

### 2.3. Manual Segmentation of the MR Images

An in-house GUI portal was developed using MATLAB R2020a (MathWorks, Natick, MA, USA) for manual delineation of tumor boundaries from the DICOM MR images. Four experts with experience in drawing ROIs on preclinical MR images were selected to annotate the imaging data. The readers were instructed to delineate the boundaries of the tumor from the T2w MR images axially through sections of the tumor as deemed by the expert. Each of the readers annotated all the test cases in a single continuous setting and identical display device and lighting conditions were used for each reader. Labels annotated by one expert were used as ground truth for training of the neural network, while the labels delineated by four experts were used to test the performance of the network. For test–retest, the tumor boundary delineation was performed by the same set of experts within a one-week gap under identical conditions.

### 2.4. CNN Model for Automatic Segmentation

#### 2.4.1. Network Architecture

A fully CNN-based on U-Net architecture was implemented to generate the segmentation maps from the PDX MR images using Keras and TensorFlow framework written in Python. The basic U-Net [18] architecture combines a down-sampling (encoder) path to capture the contextual information followed by a symmetrical up-sampling (decoder) path for accurate localization of the features. To increase the amount of contextual information in the up-sampling path, skip connections [29] are implemented to directly concatenate the feature maps from the encoder to the decoder portion of the network. Our implemented model utilized recurrent convolutional layers (RCL) [20] with two time steps, i.e., it performed two subsequent recurrent convolutions and additions following the regular convolution layer. It also used the residual connections for direct addition of the previous layer’s output to alleviate the vanishing gradient problem [21]. Dense interconnections were applied to facilitate direct concatenation of the previous layer’s information into current layer output, enhancing feature reuse [22]. 

**Figure 2:**
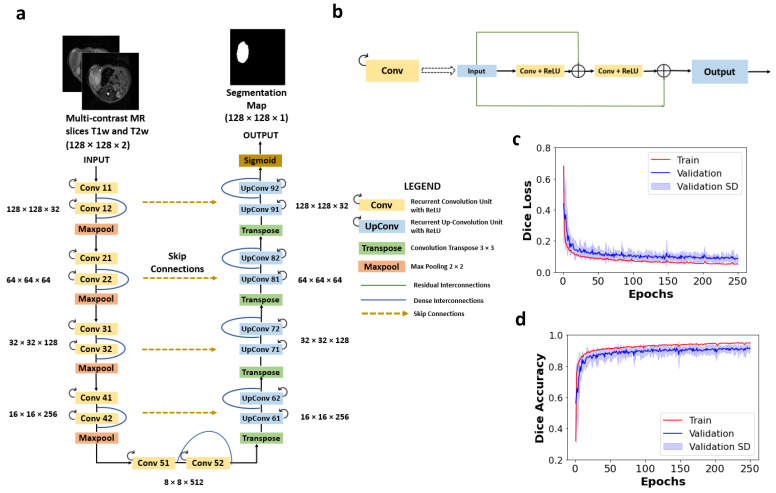
(**a**) Dense recurrent residual U-Net (D-R2UNet) architecture used for segmentation, (**b**) recurrent convolutional layer (RCL) unit of dense R2UNet, (**c**) model training and validation loss curve, (**d**) model training and validation accuracy curve for fivefold cross-validation. The validation standard deviation is shown in purple.

Convolutional layers used kernels of size 3 × 3 with a max pooling operation of 2 × 2 for detection of multiscale features in the encoder portion. Deconvolutional layers of kernel size 3 × 3 were used in the decoder portion of the network. Activation layers after each convolution operation were set as non-linear rectilinear activation units (i.e., ReLU) and a sigmoid function was used for the final activation function, setting the network’s output in the range of 0 and 1. In order to mitigate the effect of overfitting of the network due to the small dataset size available for training, spatial dropouts were implemented. The dropout layers [30] were applied prior to the max pooling in the deeper layers of the network in the main architecture and between the RCL blocks to force the network to efficiently learn the finer image features without overfitting to the peculiarities of just the training data. In all, five network architectures were tested: U-Net, dense U-Net, Res-Net, recurrent residual UNet (R2UNet), and dense R2U-Net (D-R2UNet). The architectures of the D-R2UNet and the recurrent convolutional layer unit are depicted in Figure 2a,b respectively.

#### 2.4.2. Preprocessing and Training of the Network

All input images were normalized such that the intensity distribution had zero mean and unit standard deviation for consistent CNN processing. Data augmentation was performed to make the network more robust against the degree of enlargement, rotation, and parallel shift. Each image was rotated 90°, 180°, and 270° horizontally and vertically, shifted by a factor of 0.05 and a shear range factor of 0.05. In order to perform multi-contrast segmentation and to evaluate the training efficacy, we used two images (i.e., T1w and T2w) concatenated together as channels of a single image, i.e., the network takes a single, 3-dimensional tensor (image dimension, image dimension, and channel). A fivefold cross-validation was performed on the training dataset. The dataset was split into five parts and each part was utilized for training and validation. The training set was used to train the network while the validation set was used to monitor the effectiveness of the training and fine-tuning of the hyperparameters to prevent the network from overfitting to the training data. The mean training and validation loss across the fivefold cross-validation curve is depicted by Figure 2c and the mean dice accuracy curve is depicted in Figure 2d. The validation standard deviation is also depicted in Figure 2c,d by the purple shaded region.

The training was performed on a standalone workstation equipped with a Quadro P8000 (NVIDIA) graphics processing unit. The networks were trained using the stochastic gradient descent Adam optimizer method [31] with a fixed learning rate of 1 × 10^5^. The initial weights of the filters were initialized using Xavier initialization [32]. The F1-score was used as an accuracy measure for testing the network performance during training and the dice loss was used for the loss function, which was back-propagated through the CNN for the update of the weights after each epoch. The models were trained for 250 epochs and the segmentation probability maps were obtained. In order to obtain the optimized threshold for maximizing the F1-score of the predicted segmentation, we ran the training data through the trained network to generate precision and recall curves. The intersection of the curves gives the optimum threshold value, which maximizes segmentation performance by giving the highest true positives and lowest false positives. The D-R2UNet takes approximately 3.5 h to train for 250 epochs and 100 s to make predictions on the testing dataset.

#### 2.4.3. STAPLE Algorithm to Generate Consensus among Experts

**Figure 3:**
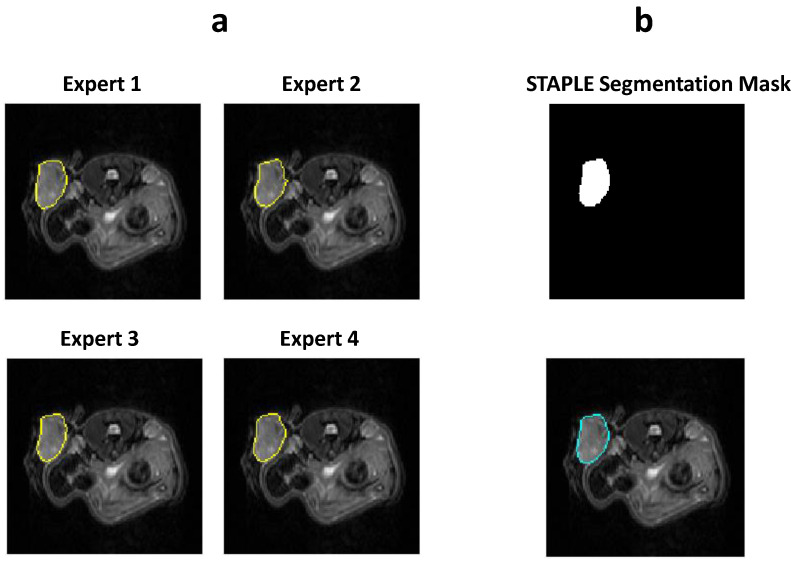
(**a**) Segmentation maps delineated by four manual experts, (**b**) segmentation map generated by applying the STAPLE algorithm to the manually delineated expert map represented in T2w image.

The STAPLE (simultaneous truth and performance level estimation) [33] algorithm was applied to compute the probability estimate of the true segmentation by simultaneously measuring performance level of each segmentation from a collection of raters using the expectation maximization algorithm. The STAPLE algorithm helps in creating a consensus map, taking into account the variability among the individual experts. The segmentation mask obtained from applying the STAPLE algorithm to the expert-generated masks was used for the assessment of the DL algorithm. Out of the 49 PDX scanned in the study, image data from 41 mice were used to train, validate, and optimize the hyperparameters of the network and image data from 8 mice were used for testing the network performance and for further radiomic analysis of the tumor region. The overview of the data is summarized in Table 1. The STAPLE estimation and the actual manual delineations are depicted in Figure 3.

#### 2.4.4. Performance Assessment of the Network

We evaluated the performance of the model in predicting tumor boundaries by using an independent testing dataset. The segmentation performance was calculated before post-processing the tumor segmentation maps by removing all but the largest continuous segmentation regions in each 2D slice for radiomic analysis. The segmentation performance of the UNet [18], Res-Net [21], DenseU-Net [23], and D-R2UNet algorithms were assessed relative to STAPLE maps. The following performance metrics expressed in terms of true positive (TP), true negative (TN), false positive (FP), and false negative (FN) were compared:

- F1-score—the F1-score measures the spatial overlap between the predicted image and the ground truth and is given by Equation (1).
  (1) $$F1\;Score=\frac{2\;TP}{2\;TP+FP+FN}\;$$
- Precision—precision signifies the fraction of true positives (TP) in relation to that of the segmented tumor region by the algorithm and is given by Equation (2).
  (2) $$Precision=\frac{TP}{TP+FP}$$
- Recall—recall signifies the fraction of true positives (TP) in relation to that of the ground truth segmentation by experts and is given by Equation (3).
  (3) $$Recall=\frac{TP}{TP+FN}$$
- Accuracy—accuracy signifies the fraction of correctly classified voxels in relation to that of the total number of voxels and is given by Equation (4).
  (4) $$Accuracy=\frac{TP+TN}{TP+FP+TN+FN}$$

### 2.5. Extraction of Radiomic Features and Correlation between STAPLE and D-R2UNet

The segmented maps obtained from the CNN along with the manually delineated maps of the experts obtained from STAPLE were arranged to create a 3D volume to perform the radiomic analysis. The radiomic features were extracted using an in-house developed program written in MATLAB (R2020a) based on the publicly available “Radiomic-Develop” repository [34] following ISBI [35]. In total 144 radiomic features were extracted for both T1w and T2w MR images. Features were divided into morphological, statistical, and histogram features, as well as GLCM, GLRLM, GLSZM, GLDZM, NGLDM, and NGTDM and were generated from 3D segmented tumor regions with 26-voxel connectivity (see Supplementary Material 1). Shape-based features were extracted from the 3D segmented volume, the intensity-based features (i.e., the first order statistics) were directly extracted from the intensity matrix generated directly from the tumor segmentation maps. Sixty-four-level fixed bin grey quantization was used to extract the histogram analysis of first order statistics. For the texture-based higher order features, a 64-level quantization was performed using the Lloyd–Max quantization algorithm [36], which iteratively calculates the optimum quantizer level and interval by utilizing the principle of probability density function. Four features were excluded as they directly correlated to some other features, like Compactness 1, Compactness 2, and Spherical Disproportion are correlated to Sphericity, while Sum Average is correlated to Joint Average [37].

The Spearman correlation coefficient (SCC, ρ) was used to determine the degree of correlation between radiomic features extracted from the STAPLE algorithm and the automated D-R2UNet algorithm. All correlation values with *p* ≤ 0.05 were considered significant. This process was repeated for both T1w and T2w and a threshold of ρ > 0.9 was determined to assess which radiomic features showed high correlation between the STAPLE and D-R2UNet maps.

### 2.6. Reproducibility of Radiomic Features by Experts

The reproducibility of segmentation and radiomic features was characterized by test/retest of manual segmentation. Test–retest of tumor boundary delineation was performed by the experts within a one-week gap in an identical setting on a randomly shuffled version of the same test dataset to check for the reproducibility. After the retest delineation, the STAPLE algorithm was applied to the segmentation maps to generate a single probability estimate map for multiple experts and it was used for reproducibility analysis of radiomic features. To test the reproducibility of the network, the model was retrained using a randomly reshuffled training and validation dataset with identical hyperparameters and then tested on the same dataset as that of the experts. Bland–Altman (BA) analysis was performed on the tumor volumes between the test–retest measurements obtained from the STAPLE algorithm (i.e., the experts) and the D-R2UNet algorithm to infer the degree of agreement between test–retest. The reproducibility of the radiomic features for both the STAPLE and D-R2UNET segmentations was investigated using the concordance correlation coefficient (CCC) [38,39].

### 2.7. Sensitivity of Features to Tumor Probability Boundaries

To evaluate the sensitivity of the features to change in tumor boundaries, we computed the SCC of the change in feature values (ΔFeature) to that of the difference in volume (ΔV) between STAPLE and D-R2UNet maps. A hierarchical clustering of the features was performed using complete linkage based on the correlation of the differences of the feature values with respect to volume change. We also extended the correlation analysis and clustering to assess the sensitivity of the radiomic features to each other i.e., cross-correlation subject to boundary change. Complete linkage was chosen for the clustering due to its highest cophenetic correlation coefficient. All of the above-mentioned statistical analyses were performed using Python 3.0 and MATLAB 2020b.

## 3. Results

### 3.1. Performance of CNN Segmentation 

The measure of agreement between the automated segmentation maps obtained from four different networks, i.e., U-Net, dense U-Net, residual U-Net (Res-UNet), and the proposed D-R2UNet relative to that of the STAPLE maps generated from the expert delineation are summarized in Table 2. The performances of the networks are given in terms of DSC, precision, recall, and AUC.

**Figure 4:**
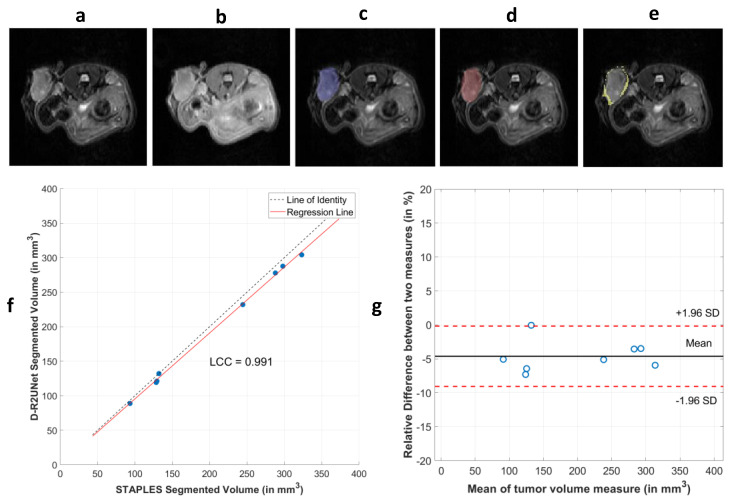
Results from the CNN segmentation by using multi-contrast MR imaging: (**a**) single slice of T2w image, (**b**) single slice of T1w image, (**c**) segmentation map derived from all the manual experts by using a EM-based algorithm called STAPLE (ground truth), (**d**) segmentation map generated by D-R2UNet, (**e**) the difference map of the D-R2UNet (algorithm) relative to the STAPLE(manual) (**f**) Lin’s concordance correlation plot between the tumor volume segmented from the D-R2UNet algorithm in relation to that of STAPLE, (**g**) BA plot between the tumor volumes segmented by D-R2UNet vs. STAPLE. The relative difference is expressed in percentage relative to ground truth and mean volume change is 4.6%.

The segmentation performance varied for single-channel (T2w only) and multichannel input (T1w and T2w). Multichannel input exhibited marginally better performance when compared to single-channel input. Among the different networks tested for multichannel input, the D-R2UNet with dice loss as loss function exhibited a better performance in terms of its mean F1-score of 0.948 (95% CI, 0.939–0.956) with respect to the other networks. The D-R2UNet also exhibited the highest precision value, signifying its efficiency in decreasing the number of false positives in detection. From the fivefold cross-validation of the different networks, D-R2UNet exhibited a greater mean F1-score, as depicted in Table 3, and thus was selected as an optimal model for further analysis. The F1-score was chosen as the primary metric for model selection and evaluating segmentation performance because it is regarded as a harmonic mean between precision and recall. Representative examples of T1w and T2w images are given in Figure 4a,b, respectively, with a STAPLE-generated map in Figure 4c. The performance of the D-R2UNet-generated segmentation map with respect to the STAPLE-generated map is depicted in Figure 4d, while the segmentation error of the D-R2UNet relative to the STAPLE algorithm is depicted in Figure 4e.

The segmented tumor volumes were used to construct the 3D tumor volume after post-processing the largest continuous area from each slice. The tumor volume extracted from the experts and the automated algorithm exhibited a high degree of correlation with CCC equal to 0.991, as depicted in Figure 4f. The BA analysis was also performed between the STAPLE and D-R2UNet algorithm tumor volume, where the bias was calculated as the difference of expert volume to that of algorithm delineated volume. The mean bias of 4.6% was obtained from the BA analysis, signifying that the algorithm underestimated the tumor volume by a mean of around 4% relative to STAPLE, which is shown in Figure 4g. The tumor volumes extracted from the D-R2UNet algorithm segmentation are correlated with the tumor volumes STAPLE-contoured by the experts (ρ = 0.99, *p* < 0.001). From the BA analysis, we also inferred that the mean tumor volume between the STAPLE and D-R2UNet was negatively correlated to the bias, with a correlation value ρ= −0.739. This means that the bias between the STAPLE and D-R2UNet decreased with increase in tumor volume.

### 3.2. Robustness of Radiomic Parameters Extracted from the D-R2UNet Algorithm

**Figure 5:**
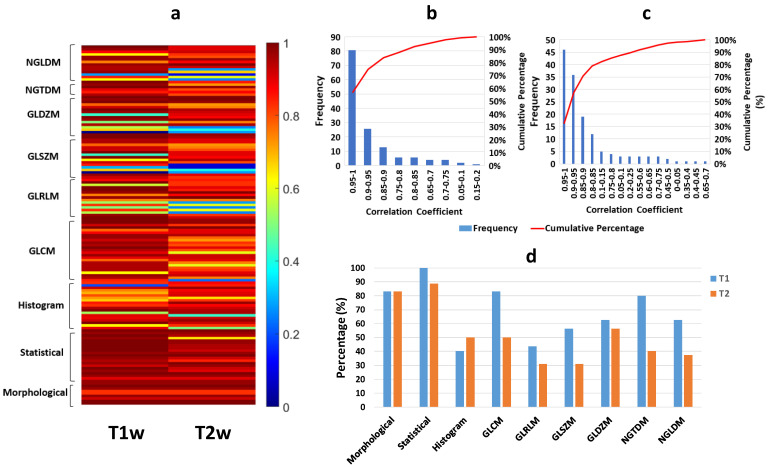
(**a**) Heatmap depicting the SCC between the radiomic features extracted from the D-R2UNet segmentation maps and the STAPLE-generated maps (ground truth) grouped by radiomic sub-category for T1w and T2w, (**b**) frequency of SCC between STAPLE and D-R2UNET for T1w, along with the cumulative sum percent of features in each binning range, (**c**) frequency of SCC between STAPLE and D-R2UNet for T2w, along with the cumulative sum percent of features in each binning range, (**d**) percentage of radiomic features that are highly correlated, i.e., ρ ≥ 0.9 (*p* ≤ 0.05) grouped by feature sub-category.

Despite the high correlation between the STAPLE- and D-R2UNET-derived tumor volumes, the extracted radiomic parameters varied significantly due to the difference in tumor boundaries. The SCC (ρ) between the STAPLE- and D-R2UNET-algorithm-generated segmentations are reported separately for each category of radiomic features and are provided in Supplementary Material 2. All 12 morphological features (shape-based) showed a high degree of correlation (0.83 ≤ ρ ≤1, *p* ≤ 0.05). The global intensity features showed a consistently high degree of correlation (0.95 ≤ ρ ≤1, *p* ≤ 0.05) for T1w and high to moderate correlation for T2w (0.66 ≤ ρ ≤ 1, *p* ≤ 0.05). For the histogram-based intensity, the degree of correlation varied significantly for both T2w (0.45 ≤ ρ ≤ 0.97, *p* ≤ 0.05) and T1w (0.69 ≤ ρ ≤ 0.97, *p* ≤ 0.05) because of the feature value’s dependence on the binning process, which is sensitive to segmentation boundaries. The correlation for the textural features also varied widely across the parameters due to the binning, with GLCM (0.61 ≤ ρ ≤ 1, *p* ≤ 0.05), GLRLM (0.83 ≤ ρ ≤ 1, *p* ≤ 0.05), GLSZM (0.73 ≤ ρ ≤ 1, *p* ≤ 0.05), GLDZM (0.73 ≤ ρ ≤ 1, *p* ≤ 0.05), NGTDM (0.8 ≤ ρ ≤ 1, *p* ≤ 0.05), and NGLDM (0.73 ≤ ρ ≤ 1, *p* ≤ 0.05). Textural features that exhibited ρ < 0.7 were found to be statistically insignificant, and hence were not considered. Figure 5a depicts the heatmap representing the degree of correlation between the D-R2UNet- and STAPLE-generated maps for each subcategory of features. A correlation ρ > 0.8 is generally considered an indication for strong correlation [40] between radiomic features and STAPLE, but we opted for a stricter threshold of ρ > 0.9 due to the relatively smaller size of our study dataset. Figure 5b,c depicts the distribution of the percentage of features from each class having high correlations. Percent of radiomic features exhibiting a correlation ρ ≥ 0.9 by class of features is depicted in Figure 5d. 

### 3.3. Reproducibility Analysis of the Radiomic Parameters

**Figure 6:**
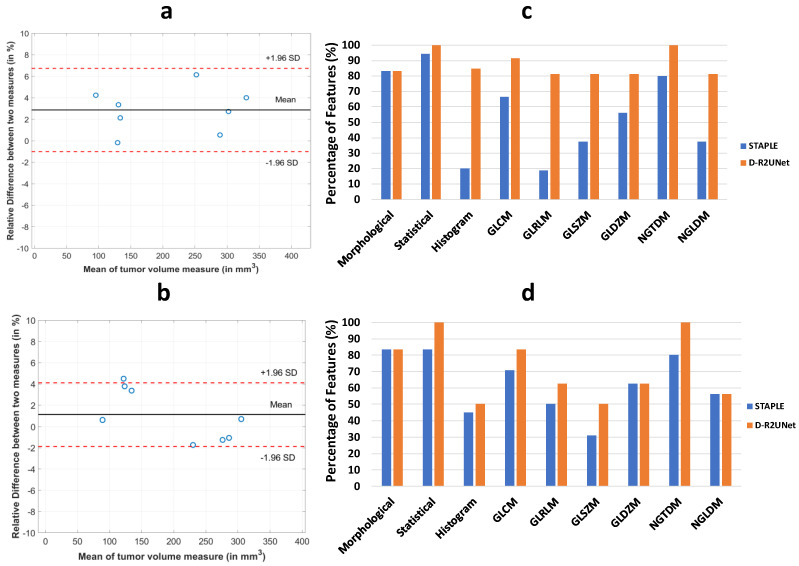
Reproducibility analysis of the D-R2UNet relative to the STAPLE: (**a**) Bland–Altman plot for test–retest for the expert delineation after application of STAPLE. The experts delineated the same set of tumor volume within a one-week gap under identical conditions. (**b**) Bland–Altman plot for test–retest for the D-R2UNet algorithm. The algorithm was re-trained in randomly shuffled data and tested on the same test data to evaluate the robustness of the algorithm. (**c**) Percentage of T1w radiomic features having CCC ≥ 0.9 extracted from STAPLE generated maps and automated maps (**d**) Percentage of T2w radiomic features having CCC ≥ 0.9 extracted from STAPLE-generated maps and D-R2UNet maps.

We evaluated the reproducibility of the radiomic features for both STAPLE-generated maps and for D-R2UNet-generated maps. BA analysis was performed on the test–retest tumor volumes for both cases, which calculated the agreement between two measurements. The mean bias of measurement for tumor volume for test–retest among the experts ranged from −2.2% to 8.37% relative to the first delineation. This wide range of variation was solved by using STAPLE for test–retest, which gave a mean bias of −2.8% relative to first delineation, as depicted in Figure 6a. D-R2UNet outperformed every expert and the STAPLE by achieving a mean bias of measurements equal to 1.02% relative to first training run of the D-R2UNet, as depicted in Figure 6b. The CCC were used to assess test–retest performance of radiomic features for both T1w and T2w MR images. A feature was considered to be highly reproducible if it had CCC > 0.9 [41,42]. For both D-R2UNet- and STAPLE-generated radiomic features, greater than 80% of morphological and greater than 90% of statistical features were highly reproducible. For higher order textural features, the number of reproducible features decreased due to quantization. The percent of reproducible features varied around 70–80% for T1w and 60–70% for T1w with respect to each higher order radiomic subcategory (Supplementary Material 3) and is given in Figure 6c,d, respectively.

### 3.4. Sensitivity of Radiomic Features to Tumor Boundaries

**Figure 7:**
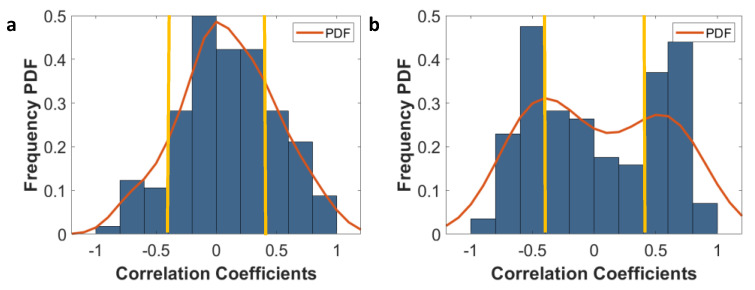
(**a**) Frequency distribution of the SCC between the change in radiomic features (ΔFeatureValue) to change in volume (ΔV) along with the underlying probability density function (PDF) of the distribution (in red) for T1w. (**b**) Frequency distribution of the SCC between the change in radiomic features (ΔFeatureValue) in relation to change in volume (ΔV) along with the underlying PDF of the distribution (in red) for T2w. The yellow line signifies the −0.4 to 0.4 interval, i.e., where ΔFeatureValues are considered insensitive to ΔVolume.

To evaluate the sensitivity of the radiomic features to change in the tumor boundaries, the SCC between changes in the radiomic features relative to the changes in tumor volume were calculated. The frequency distributions and their underlying probability density functions are depicted in Figure 7a,b for T1w and T2w, respectively. Features that had SCC values in the range of −0.4 to 0.4 were considered to be robust to perturbations. Ninety-five T1w radiomic features and fifty T2w radiomic features were found to be robust to perturbations in the tumor boundary (Supplementary Material 4). 

**Figure 8:**
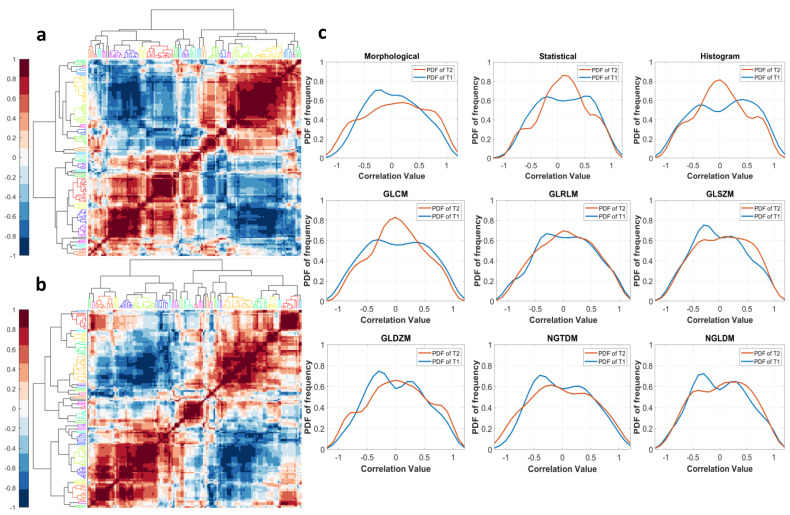
(**a**) Clustergram for correlation between the ΔFeatureValues for T1w. (**b**) Clustergram for correlation between the ΔFeatureValues for T2w. (**c**) The PDF for T1w and T2w for the distribution of the ΔFeatureValues’ Spearman correlation coefficient by radiomic sub-category.

The hierarchical clustering of the cross-correlation of change in feature values resulted in 23 and 26 independent clusters for T1w and T2w, respectively, using complete linkage and dendrogram length = 4, as depicted by the clustergram in Figure 8a,b, respectively, for T1w and T2w. The PDF for the sub-categorical distributions of cross-correlation of the change in feature values are given in Figure 8c. The features representing each independent cluster for T1w and T2w are given in Supplementary Material 5.

## 4. Discussion

In this paper, we implemented CNN-based methods for automatic segmentation of tumors in multi-contrast preclinical MR imaging. All CNN methods proposed within this study performed better than previously published preclinical tumor segmentation methods, including fast k-means-based level-set method [43], which achieved a F1-score = 0.82 in segmenting TNBC PDX MR images, and multi-contrast U-Net, which achieved a F1-score = 0.84 in segmenting sarcoma tumors in MR [44]. Of the five networks tested in the current work, DR2U-Net exhibited marginally better performance in terms of F1-score compared to other DL methods implemented in this work. The dense residual interconnections and the recurrent convolutional units (RCL) facilitated faster learning of features from limited data space and fewer parameters by gradient propagation and feature reuse.

The use of multi-contrast MR imaging instead of single contrast has significantly shown performance improvement in brain segmentation using DL [45]. Our segmentation model was also in agreement with this fact and achieved the best performance for multi-contrast data versus T2w-only data (Table 1). The multi-contrast data combined features from T1w and T2w and facilitated better learning of features. Our approach mimics the clinical scanning protocols where multi-contrast MR are used by radiologists to assess tumor boundaries. One principal issue with manual tumor delineation is the variability of delineation among multiple experts, which leads to lack of reproducibility [46,47]. In this study, as expected, the tumor volume differed substantially between the experts, demonstrating the need to develop a reproducible pipeline. Even after the application of the STAPLE algorithm, which creates a probabilistic map, taking into consensus all the expert delineations, there was variability in repeated delineations among the ground truth delineated by experts. The algorithm was found to be more robust on train–retrain measures, with only 1.02% mean volume bias between two runs. Though the training of DL networks a takes considerable amount of time and resources, it is still less labor intensive and more reproducible than manual intervention.

The fully automated method also accelerated high-throughput extraction of quantitative radiomic features, enabling extraction of mineable data from segmented tumors. Intensity and shape-based (first order) features were highly corrected between the STAPLE and D-R2UNet algorithms. However, the correlation of texture-based features between STAPLE the D-R2UNet varied widely. The textural features were more sensitive to the change in segmentation boundaries as they were extracted by intensity binning of the intensity histogram into different quantization levels. The intensity quantization levels were drastically affected due to the change in boundaries, as change by a few voxels of delineation can affect the intensity quantization process, hence affecting higher-order features. We observed that there was a greater degree of correlation for T1w features than T2w higher-order features because T2w has more variability in texture than T1w, and hence even small perturbations had greater impact on the quantization process. The reduction in number of bins would result in a higher correlation in radiomic features between STAPLE and D-R2UNet, but would fail to capture the dynamic texture of the tumor.

Reproducibility and repeatability are essential elements to enhance the translation of radiomics to clinical practice [48]. Manual delineations are particularly prone to reproducibility issues [49,50,51]. Haarburger et al. compiled a set of robust features by analyzing reproducibility of features for both manual segmentation and probabilistic automated segmentation for clinical CT images [52]. Zwanenburg et al. assessed the robustness of radiomic features to image perturbations associated with test–retest measures [53]. The CCC [41,42,54,55,56] is widely used as a metric to quantify reproducibility of the features. A suitable threshold value has not yet been established as different studies have used different thresholds. The value of CCC > 0.75 is an indicative of good reliability between two measurements [57,58]. We selected a stricter threshold of 0.9 to signify reproducibility for CCC [41,42], owing to our small number of datapoints to avoid the Type I and Type II errors. The D-R2UNet showed more consistent results with minimal volume bias of 1.02% when subjected to test–retest measures relative to the STAPLE algorithm, which had a volume bias of 2.8%. Even though the volume was is minimal, due to the sensitivity of the features, the number of reproducible features varied widely across for STAPLE and D-R2UNet algorithms. We also observed a greater number of features to be reproducible for T1w than T2w for D-R2UNet because of its less heterogenic texture. 

Since variability in delineation of tumor boundaries is inevitable, we attempted to characterize features that are less sensitive to perturbations in tumor boundaries. Among these robust features, we further characterized features that were highly correlated to STAPLE maps (ρ ≥ 0.9, *p* ≤ 0.05) and were also reproducible for D-R2UNet (CCC ≥ 0.9, *p* ≤ 0.05) (Supplementary Material 4). We found that for T1w 56 features, i.e., 36.16%, and for T2w 20 features, i.e., 13.9%, features fit all criteria. These groups of features can be used as biomarker indicators in studying treatment response in the preclinical setting using the DL pipeline.

## 5. Conclusions

In conclusion, we have implemented and tested DL-based pipelines for accurate and automatic localization of TNBC PDX in multi-contrast small animal MR imaging. DR2UNet performed marginally better than other implemented networks. Nevertheless, the automated methods ensure high throughput tumor segmentation and minimize manual intervention, which in turn enhances reproducibility. Furthermore, we have implemented a radiomics pipeline to characterize the sensitivity of the features to perturbations in tumor boundary. The automated generated maps were found to be highly correlated and reproducible relative to the STAPLE maps and thus can be used for high throughput phenotyping of preclinical MR images in co-clinical trials.

## Figures and Tables

**Table 1:** Overview of the dataset used for the network training and testing.

Total No. of Mice Used for Study	No. of Mice Used for Training and Validation of the CNN	No. of Mice Used for Testing the Performance of the CNN	No. of MR Slices for Training and Validation	No. of MR Slices Used for Testing
49	41	8	255	39

**Table 2:** Performance metrics (mean ± SD) of PDX tumor segmentation for different network models relative to STAPLE map.

Input Data	Network	F1-Score	Recall	Precision	AUC
T2w and T1w	U-Net	0.929 ± 0.072	0.928 ± 0.040	0.935 ± 0.098	0.962 ± 0.019
	Dense U-Net	0.923 ± 0.066	0.960 ± 0.025	0.897 ± 0.116	0.977 ± 0.012
	Res-Net	0.922 ± 0.074	0.947 ± 0.034	0.910 ± 0.117	0.971 ± 0.017
	R2U-Net	0.929 ± 0.072	0.933 ± 0.037	0.937 ± 0.128	0.965 ± 0.018
	Dense R2U-Net	0.948 ± 0.026	0.928 ± 0.032	0.970 ± 0.042	0.963 ± 0.016
T2w	U-Net	0.927 ± 0.076	0.950 ± 0.031	0.919 ± 0.119	0.973 ± 0.016
	Dense U-Net	0.910 ± 0.077	0.932 ± 0.033	0.900 ± 0.130	0.963 ± 0.017
	Res-Net	0.913 ± 0.079	0.884 ± 0.079	0.943 ± 0.091	0.943 ± 0.040
	R2U-Net	0.924 ± 0.067	0.959 ± 0.026	0.902 ± 0.116	0.977 ± 0.012
	Dense R2U-Net	0.935 ± 0.064	0.954 ± 0.023	0.925 ± 0.107	0.975 ± 0.011

**Table 3:** Performance metrics (mean ± SD) of fivefold cross-validation for the different network models.

Network	F1-Score	Recall	Precision	AUC
U-Net	0.906 ± 0.022	0.907 ± 0.027	0.914 ± 0.015	0.961 ± 0.013
Dense U-Net	0.911 ± 0.016	0.903 ± 0.025	0.930 ± 0.016	0.960 ± 0.012
Res-Net	0.909 ± 0.010	0.902 ± 0.030	0.925 ± 0.028	0.961 ± 0.012
R2U-Net	0.917 ± 0.013	0.909 ± 0.030	0.933 ± 0.008	0.960 ± 0.013
Dense R2U-Net	0.922 ± 0.009	0.937 ± 0.005	0.928 ± 0.016	0.963 ± 0.008

## Data Availability

Python code for D-R2UNet is available in GitHub https://github.com/WU-C2IR2/DR2UNet-for-TNBC-PDX-Tumor-Segmentation (accessed on 26 July 2021) and the co-clinical data will be available for download through the Washington University School of Medicine Co-Clinical Imaging Research Resource web portal at https://c2ir2.wustl.edu/ (accessed on 26 July 2021).

## Supplementary Materials

The following Supplementary Material are available online at https://www.mdpi.com/article/10.3390/cancers13153795/s1, Supplementary Material 1: Radiomic feature descriptions, Supplementary Material 2: Spearman correlation coefficient between STAPLE and D-R2UNet maps, Supplementary Material 3: Reproducibility analysis by CCC, Supplementary Material 4: List of the features that are robust to tumor boundary perturbations, Supplementary Material 5: List of features in each individual clusters formed by the cross-correlation of change in feature values.
